# EXAMINING AGE BIAS IN CHATGPT RESPONSES TO USER QUESTIONS ABOUT DECISION-MAKING IN ADVANCED CANCER

**DOI:** 10.1093/geroni/igae098.2386

**Published:** 2024-12-31

**Authors:** Meghan McDarby, Emily Mroz, Jessica Hahne, Brian Carpenter, Patricia Parker

**Affiliations:** Memorial Sloan Kettering Cancer Center, New York, New York, United States; Emory University, Decatur, Georgia, United States; Washington University in St. Louis, St. Louis, Missouri, United States; Memorial Sloan Kettering Cancer Center, New York, New York, United States; Memorial Sloan Kettering Cancer Center, New York, New York, United States

## Abstract

ChatGPT is an online, AI-powered chat application that provides human-like responses to user input. There is concern about bias in ChatGPT responses based on user demographics, yet no existing research has examined age bias in ChatGPT responses to questions about decision making (DM) in advanced cancer. The current study examined the content of ChatGPT responses to a hypothetical patient question about DM in advanced cancer. Replicating Nastasi and colleagues’ (2023) approach, we posed a medical-advice-seeking vignette to ChatGPT with variations on four hypothetical patient characteristics: age (45/65/85 years), race (Black/White), ethnicity (Hispanic/Non-Hispanic), and insurance status (Insured/Not Insured). Each vignette ended with: “Should I look for other treatments or focus on my quality of life?” Three coders categorized responses (ICC=.95) for mentions of nine topics relevant to DM in advanced cancer (e.g., consideration of clinical trials, suggestion to pursue second opinions, descriptions of hospice and palliative care, and encouragement to talk to providers for DM support). ChatGPT responses more frequently mentioned consideration of clinical trials to patients who were 45 compared to patients who were 65 and 85 years old (X2(2,N=96)=8.6, p=.01). There was a non-significant trend toward ChatGPT responses suggesting pursuit of second opinions more frequently for younger patients (X2(2,N=96)=3.0 p=.22). There were no age differences in mentions of hospice care (p>.50), palliative care (p>.30), or talking to providers for DM support (p>.30). Findings indicate that ChatGPT responses to questions about advanced cancer DM may contain age bias. Future research should address how ChatGPT users navigate bias in responses.

